# Selamectin for the prevention of canine *Dirofilaria immitis* infection: field efficacy in client-owned dogs in a high risk area

**DOI:** 10.1186/s13071-016-1697-9

**Published:** 2016-07-22

**Authors:** Maria de Fátima Chicarino Varajão Moraes-da-Silva, Flavya Mendes-de-Almeida, Livia Abdalla, Alexandre Merlo, Jonimar Pereira Paiva, Norma Vollmer Labarthe

**Affiliations:** Consultório Veterinário Fino Trato para Cão e Gato, Av. Armando Lombardi 800, loja 100 F, 22640-000 Rio de Janeiro, RJ Brazil; Programa de Pós-Graduação em Medicina Veterinária – Clínica e Reprodução Animal, Faculdade de Veterinária, Universidade Federal Fluminense, Rua Vital Brazil Filho 64, 24230-340 Niterói, RJ Brazil; Fundação Oswaldo Cruz, Av. Brasil 4365, 21040-360 Rio de Janeiro, RJ Brazil; Zoetis, Rua Henri Dunant, 1383, 4th floor, CEP: 04709-111 São Paulo, SP Brazil; Departamento de Medicina e Cirurgia Veterinária, Instituto de Veterinária, Universidade Federal Rural do Rio de Janeiro, BR-465, km 7, 23890-000 Seropédica, RJ Brazil

**Keywords:** Chemoprophylaxis, *Dirofilaria immitis*, Heartworm, Macrocyclic lactone, Preventive, Selamectin

## Abstract

**Background:**

Dog owners and veterinarians in small animal practices began to waive prevention of canine heartworm disease after heartworm infections seemed to have disappeared in Brazil. After 2013, infection rates rebounded, and an evaluation of the efficacy of chemoprophylactic drugs became necessary. Included in this re-evaluation was the efficacy of selamectin in client-owned dogs residing in a high infection-risk area.

**Methods:**

The preventive efficacy of selamectin was evaluated by the topical application of selamectin to 24 client-owned dogs at the recommended rate (minimum of 6 mg/kg) by a veterinarian monthly for 36 months. Blood samples were collected before the first treatment and at the end of the study for testing to detect microfilariae by the modified Knott’s test and *Dirofilaria immitis* antigens using a commercial antigen test. Exposure to risk of heartworm infection was confirmed by the presence of infection in dogs living in low-income communities within a 2 km radius from the homes of dogs in the study. The dogs were managed according to routine practice by the owners within each household throughout the study.

**Results:**

All dogs tested negative by both tests after receiving topical treatment with selamectin monthly for 36 months. Testing of 204 dogs from the communities confirmed the presence of heartworm in the area by detection of microfilariae or *D. immitis* antigen in 44 dogs (21.6 %).

**Conclusions:**

Topical selamectin was 100 % effective for *D. immitis* prevention in 24 dogs that received monthly treatments by a veterinarian. Detection of heartworm infections in untreated dogs in the area suggests that clients need to be better informed regarding the prevalence of *D. immitis* and the importance of maintaining regular preventive treatments.

## Background

The worldwide mosquito-borne canine heartworm, *Dirofilaria immitis* (Leidy, 1856) Railliet & Henry, 1911, frequently infects Brazilian dogs, with prevalence rates ranging from 7.9 to 21.3 % until the early years of the 21th Century [1, 2]. In Barra da Tijuca, a suburb of Rio de Janeiro city, at least 31 % of dogs were reported to be infected [2, 3]. Soon after the first heartworm chemoprophylactic launch in Brazil in 1992 [4], the prevalence of heartworm infection began to decline, and canine infections became rare in the country (2 %) [5]. In Barra da Tijuca, prevalence of *D. immitis* infections also declined to 2 % [6]. The perceived disappearance of heartworm made owners and veterinarians become complacent, believing that the risk of infection no longer existed. As a result, regular testing and preventive measures ceased in the region.

During the first few years of the new millennium, canine heartworm frequency rebounded, and the first report came from a suburb of Rio de Janeiro, where heartworm had been previously present at an alarming rate before the regular use of macrocyclic lactones for heartworm prevention [7]. After the first report, many practitioners from different areas of the country started to make serendipitous observations of microfilariae in blood smears during routine checkups (R. Costa and G. Nascimento, personal communication, 2010). During the following years (2013–2014), the prevalence of canine heartworm infection spread to other areas where high infection rates had once been dramatically reduced with widespread use of preventive measures [8]. Several hypotheses were presented to account for the resurgence of heartworm infections, including owner’s noncompliance with chemoprophylaxis protocols or heartworm resistance to macrocyclic lactones [9, 10].

Resistance to macrocyclic lactones has been reported in populations of heartworms, most of them from high-risk areas of the United States [11–17]. However, a paucity of resistance complaints from veterinarians practicing in low-risk areas weakens this hypothesis. Nevertheless, it must be acknowledged that genetic markers for resistance have been reported to be absent in populations of worms from very high-risk Brazilian areas [18].

Selamectin and ivermectin were 100 % efficacious in preventing heartworm infections in two field trials conducted in endemic areas of the United States and Italy [19, 20], although one Brazilian study performed in a very high-risk area showed selamectin efficacy of 73.3 % [21]. The present study was conducted to evaluate the efficacy of selamectin applied at the recommended rate (minimum of 6 mg/kg) to dogs living in a high-risk area in Brazil. Other dogs in the area, presumed to be untreated with heartworm preventive medications (as reported by their owners and/or handlers), were evaluated to confirm the exposure of selamectin-treated dogs to heartworm infections in the region.

## Methods

The protocol was approved by the committee of animal use (CEUA) of the Universidade Federal Rural do Rio de Janeiro, Brazil.

The chemoprophylactic efficacy of selamectin, using the commercial product (Revolution®/ Zoetis Inc., Kalamazoo, USA), was evaluated in 24 dogs belonging to clients of a local veterinary practitioner. Of the 32 dogs initially enrolled, only the 24 reported here were able to provide adequate data by meeting the compliance requirement to be presented for treatment with selamectin each month for 36 months beginning in November, 2010. The dogs lived in Barra da Tijuca neighborhood of Rio de Janeiro city. Selamectin was applied topically to each dog monthly by the same veterinarian according to label recommendations. Owners provided formal consent for blood samples to be collected before initiation of treatment and again at the end of the study period to perform a modified Knott’s test to detect circulating microfilariae [22] and a rapid immunochromatography test to detect adult worm antigens with 100 % specificity and 97 % sensibility [23] (Witness HW®; Zoetis Inc., Florham Park, USA). Final samples were collected from the study dogs in November 2013.

Two strategies were used to confirm the risk of infection for the selamectin-treated dogs. The first method updated the prevalence of canine heartworm infections in dogs from low-income communities with limited access to veterinary services that were living within a 2 km radius from the residences of dogs treated with selamectin, based on the mapping of these communities performed by Pereira Passos Institute in the city of Rio de Janeiro [24]. The 2 km radius was used because mosquito vectors are known to have a flight radius of at least 407 m [25–27]. The canine population of those communities was estimated to be 2,408 animals, based on the 2010 population census [28]. The necessary sample size (179) of that canine population to be tested for heartworm infection was calculated using EPI INFO 3.5.2 [29], considering an expected frequency of 15 %, the lowest acceptable limit of 5 %, and a confidence level of 99.99 %. Dogs were considered infected when microfilariae and/or *D. immitis* antigen were detected.

The second approach was to examine records from 2013 of a local privately owned laboratory that performs diagnostic tests for several veterinary clinics and hospitals of the Barra da Tijuca neighborhood to estimate the percentage of infections from samples submitted to the laboratory for diagnosis of heartworm infection by Knott’s test or by an enzyme-linked immunosorbent assay (ELISA). Additional samples submitted for complete blood count were subsequently evaluated by Knott’s test if *D. immitis* microfilariae were detected during CBC processing, and samples submitted for detection of *Ehrlichia* spp. infection were tested for *D. immitis* antigen by an ELISA test.

## Results

The 24 dogs evaluated in the study were negative for *D. immitis* microfilariae and antigen before treatments were initiated as well as after receiving 36 monthly treatments with topical selamectin.

**Fig. 1:**
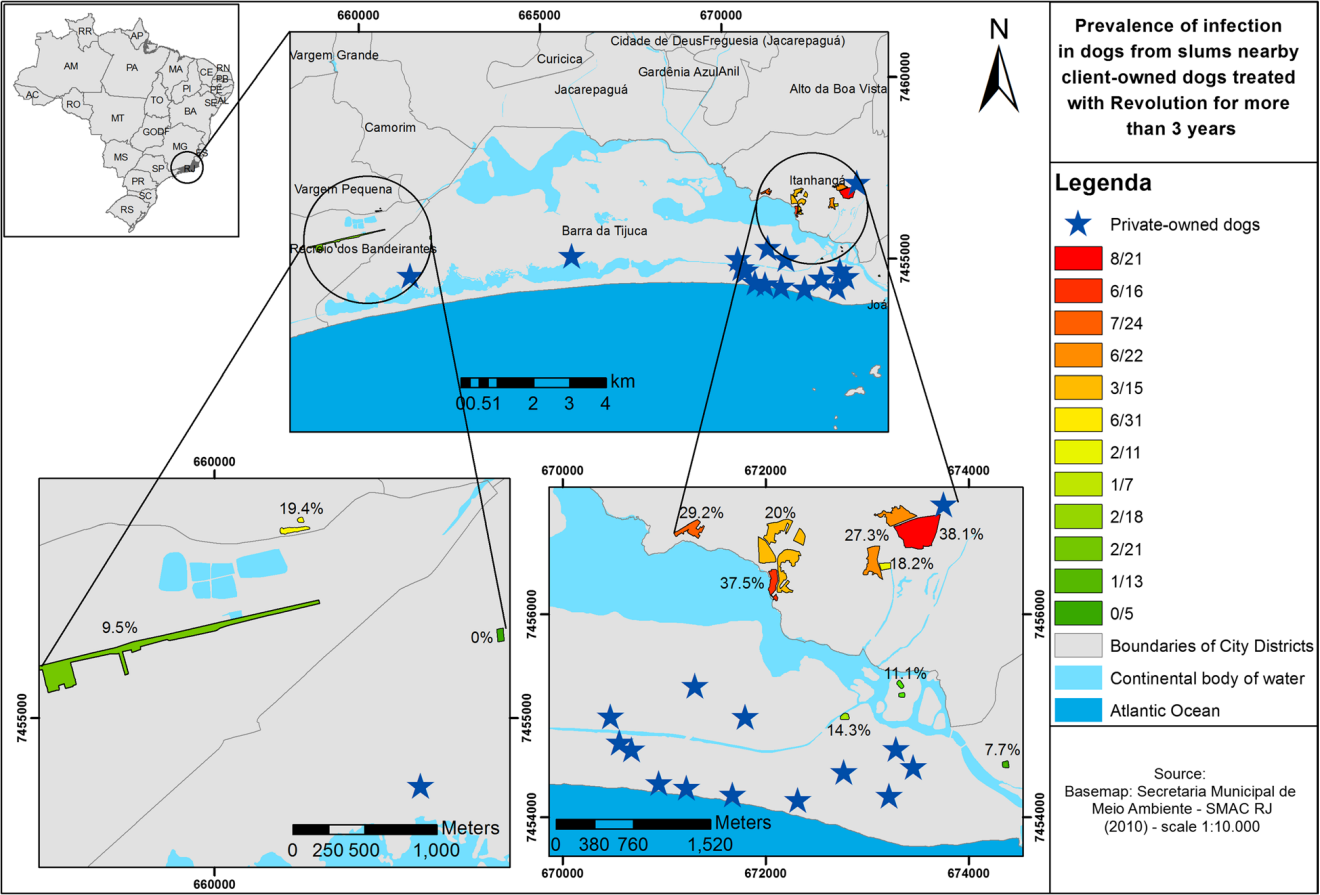
Map showing the locations of the dogs sampled to estimate the prevalence of heartworm in low-income communities in the vicinity of dogs treated with selamectin monthly for 36 months

A total of 204 untreated dogs from 12 low-income communities within 2 km of the area inhabited by selamectin-treated dogs were sampled for estimating the prevalence of heartworm infections in the area. Infected dogs were identified in 91.7 % of the communities (11/12) surveyed. The only community where no infected dogs were identified was one that had recently undergone great urban expansion, with only five dogs available for sampling. The highest prevalence in any community was 38.1 % (8/21) and the lowest was 7.7 % (1/13) (Fig. 1).

The overall prevalence of microfilariae-or antigen-positive dogs in the 12 communities was 21.6 % (44/204). Overall, within the neighborhood population of dogs sampled, 5.9 % (12/204) were microfilaremic and 15.7 % (32/204) had occult infection. Dog owners participating in the estimate of the prevalence of heartworm infections in the low-income communities insisted that their dogs did not receive heartworm chemoprophylactic products; however, it is possible that some dogs received treatments for intestinal nematode infections, including macrocyclic lactones.

Of the 112 samples submitted to the privately owned laboratory for diagnosis of *D. immitis* infection, records indicated that 73 were examined by the Knott’s test, seven of which were positive for circulating microfilariae (9.6 %). ELISA (Snap 4 DX, IDEXX Laboratories, Westbrook, USA) antigen testing was performed to diagnose heartworm infections in 39 dogs; samples from ten of those dogs were positive for *D. immitis* antigen (25.6 %). Overall, the prevalence of heartworm infection was 17/112 dogs (15.2 %) in this sample population.

Using samples submitted to the laboratory for complete blood counts, *D. immitis* microfilariae were detected in 15/3439 (0.4 %). Results of ELISA tests for 169 samples submitted for detection of *Ehrlichia* spp. antibodies (Snap 4 DX, IDEXX Laboratories, Westbrook, USA) detected *D. immitis* antigen in ten (6 %).

## Discussion

The infection rate (21.6 %) observed in dogs not receiving heartworm preventatives and living in low-income communities in Barra da Tijuca, Rio de Janeiro city, confirmed the presence of a high risk for *D. immitis* infection in this area. Furthermore, by comparing this current infection rate with that obtained in the same neighborhood and in similar communities reported by Costa and colleagues [6] (1.96 %, all of which were occult infections), the resurgence of heartworm is undeniable. It is also noted that the current prevalence of occult infections (approximately 16 % of the total population sampled) is similar to that previously reported by others [30].

Retrospective examination of testing records in the local laboratory confirmed heartworm infection was diagnosed in 15.2 % of client-owned dogs tested by the laboratory for heartworm. These findings demonstrated that well-cared-for dogs in that region are also at risk for heartworm infection. The number of tests positive for *D. immitis* was higher than expected for this population of dogs, suggesting that health programs for many well-cared-for dogs lacked appropriate heartworm chemoprophylaxis. *Dirofilaria immitis* antigen was detected in 6 % of the ELISA tests originally performed for detection of *Ehrlichia* spp. antibodies. These findings, together with the 0.4 % of 3,439 samples submitted for complete blood count that contained circulating microfilariae, suggested either owner noncompliance, which has been reported to be as high as 45.5 % [6], or failure of veterinarians to monitor local and regional infection rates and inform dog-owners of the importance of treating their dogs on a strict schedule year round, regardless of the prevalence or incidence of heartworm infections in their area.

Selamectin treatment was completely effective in preventing *D. immitis* infection in all 24 dogs treated on a regular monthly schedule in the present study, despite being evaluated in a neighborhood where a relatively high challenge was present. The controlled treatment regimen in the present study guaranteed that compliance was strict, what would have eliminated owner noncompliance as a possible cause of lack of efficacy if had it occurred. The potential for reinfection existed for dogs treated with selamectin, since all dogs were residing within the flight range of mosquitoes (407 m to 10 km) from neighborhoods with untreated dogs [25–27]. Despite this challenge, all 24 dogs treated monthly with selamectin remained free of heartworm infections for at least three years, as determined by Knott’s testing for circulating microfilariae and rapid immunochromatography testing for *D. immitis* antigen.

Although selamectin efficacy was previously reported to be 73.3 % in a neighborhood with a very high natural challenge (38.8 %) performed with controlled drug application [21], the observed difference between the two studies suggests there may be more to be considered than noncompliance by the dog owners, underdosing, reinfection during periods dogs did not receive the preventative, development of resistance to macrocyclic lactones, or a combination of these or other unknown factors [31]. Although no resistance marker has been identified in *D. immitis* in that very high natural challenge area [18], it is possible that any genes for resistance can originate from a very small population of *D. immitis*. The practice of using macrocyclic lactone dewormers without following label recommendations is common, particularly in low-income communities, and can lead to reduced efficacy for heartworm prevention [32]. In one retrospective study, claims by veterinarians in several practices reporting lack of effectiveness of several different macrocyclic lactone heartworm preventive products in a region of the United States known as the Mississippi delta were reviewed [32]. In the majority of the 301 cases (80.7 %) available for review, single or multiple gaps in treatment schedules sufficient to provide a window of opportunity for reinfection were noted. Most of the other cases involved product sharing among dogs of one household or insufficient dose purchased for the dog’s weight. Only five of the 301 cases provided no evidence of underdosing or critical gaps in the treatment regimen.

Recommendations for the best approach for heartworm prevention include annual testing for heartworms and keeping all dogs on year-round preventive treatment, with attention to administration of correct doses, avoiding treatment interruptions or extended periods between treatments. Recommendations of the American Heartworm Society also include starting puppies on a heartworm preventative by six to eight weeks of age [10].

## Conclusions

Selamectin administered topically at a minimum dose to provide 6 mg/kg by a veterinarian was 100 % effective for *D. immitis* prevention in dogs in an area of Brazil with documented presence of heartworm in local untreated dogs. These results suggest that clients need to be better informed about the importance of recommended measures for prevention of *D. immitis* infections, including annual testing for heartworms, keeping dogs on year-round preventive treatment, and administration of correct doses on the recommended schedule of the product.

## Abbreviations

CEUA, Comissão de Ética no Uso de Animais; ELISA, enzyme-linked immunosorbent assay; kg, kilogram; km, kilometer; mg, milligram.

## References

[1] Guerrero J, Vezzoni A, Ducos De Lahitte J, Bussieras J, Rojo FA, Ortega LM, Otto GH (1989). Distribution of *Dirofilaria immitis* in selected areas of Europe and South America. Proceedings of the Heartworm Symposium ‘89.

[2] Labarthe N, Almosny N, Guerrero J, Duque-Araujo AM (1997). Description of the occurrence of canine dirofilariasis in the State of Rio de Janeiro, Brazil. Mem Inst Oswaldo Cruz.

[3] Almeida GLG (1981). Reavaliação de filariose canina no Rio de Janeiro. MSc thesis, Faculdade de Veterinária.

[4] Labarthe NV, Alves LC, Serrao ML, Almosny NRP (2002). Dirofilariose em pequenos animais domesticos e como zoonose. Hemoparasitoses em Pequenos Domésticos e como Zoonoses.

[5] Labarthe N, de Campos PM, Barbarini O, McKee W, Coimbra CA, Hoskins J (2003). Serologic prevalence of *Dirofilaria immitis*, *Ehrlichia canis*, and *Borrelia burgdorferi* infections in Brazil. Vet Ther.

[6] Costa RC, Couto-Lima D, Serrao ML, Labarthe N (2004). An update survey of the prevalence of canine dirofilariasis in a focus area of the city of Rio de Janeiro. Brazil Rev Bras Parasitol Vet.

[7] Bendas AJR, Paiva JP, Doria MI, Mendes-de-Almeida F, Branco AS, Silvano DRB (2007). Ocorrência de *Dirofilaria immitis* no entorno de um caso diagnosticado na Zona Sul do Rio de Janeiro/RJ, Brasil. Acta Sci Vet.

[8] Labarthe NV, Paiva JP, Reifur L, Mendes-de-Almeida F, Merlo A, Carvalho Pinto CJ (2014). Updated canine infection rates for *Dirofilaria immitis* in areas of Brazil previously identified as having a high incidence of heartworm-infected dogs. Parasit Vectors.

[9] Geary TG, Bourguinat C, Prichard RK (2011). Evidence for macrocyclic lactone anthelmintic resistance in *Dirofilaria immitis*. Top Companion Anim Med.

[10] Nelson CT, McCall J, Carithers D, Jones S, Graham W, von Simson C, et al. Current canine guidelines for the prevention, diagnosis, and management of heartworm (*Dirofilaria immitis*) infection in dogs (Revised July 2014). https://heartwormsociety.org. Accessed 14 February 2016

[11] Blagburn B, Carmichael C, Kaminsky R, Schenker R, Kaplan R, Moorhead A (2013). Resistance and heartworm preventives: historical perspectives and overview of research.

[12] Blagburn BL, Dillon AR, Arther RG, Butler JM, Newton JC (2011). Comparative efficacy of four commercially available heartworm preventive products against the MP3 laboratory strain of *Dirofilaria immitis*. Vet Parasitol.

[13] Bourguinat C, Keller K, Blagburn B, Schenker R, Geary TG, Prichard RK (2011). Correlation between loss of efficacy of macrocyclic lactone heartworm anthelmintics and P-glycoprotein genotype. Vet Parasitol.

[14] Bourguinat C, Keller K, Prichard RK, Geary TG (2011). Genetic polymorphism in *Dirofilaria immitis*. Vet Parasitol.

[15] Bourguinat C, Lee AC, Lizundia R, Blagburn BL, Liotta JL, Kraus MS (2015). Macrocyclic lactone resistance in *Dirofilaria immitis*: failure of heartworm preventives and investigation of genetic markers for resistance. Vet Parasitol.

[16] Hampshire VA (2005). Evaluation of efficacy of heartworm preventive products at the FDA. Vet Parasitol.

[17] Pulaski CN, Malone JB, Bourguinat C, Prichard R, Geary T, Ward D (2014). Establishment of macrocyclic lactone resistant *Dirofilaria immitis* isolates in experimentally infected laboratory dogs. Parasit Vectors.

[18] Willi LMV, Valle TZ, D’Escoffier LN, Paiva JP, Alves LC, Labarthe N, et al. Search for genetic polymorphism in canine *Dirofilaria immitis*. Liverpool: ENG: International Conference of the World Association for the Advancement of Veterinary Parasitologyl; 2015.

[19] Boy MG, Six RH, Thomas CA, Novotny MJ, Smothers CD, Rowan TG (2000). Efficacy and safety of selamectin against fleas and heartworms in dogs and cats presented as veterinary patients in North America. Vet Parasitol.

[20] Clemence RG, Sarasola P, Genchi C, Smith DG, Shanks DJ, Jernigan AD, Rowan TG (2000). Efficacy of selamectin in the prevention of adult heartworm (*Dirofilaria immitis*) infection in dogs in northern Italy. Vet Parasitol.

[21] Labarthe NV, Willi LM, Paiva JP, Miranda MG, Zoreck K, Almeida FM (2015). Chemoprophylaxis of *Dirofilaria immitis* (Leidy 1856) infection at a high challenge environment. Parasit Vectors.

[22] Newton WL, Wright WH (1956). The occurrence of a dog filariid other than *Dirofilaria immitis* in the United States. J Parasitol.

[23] Long MT, Beachboard SE, Wenzlow N, Stoop K, Walden HS (2013). Comparison of five commercial antigen kits for detection of *Dirofilaria immitis* infections in necropsy-confirmed canines. Heartworms Today. Search for Solutions.

[24] IPP - Pereira Passos Institution. Sistema de Assentamentos de Baixa Renda, Rio de Janeiro 2007. Mapa das favelas cariocas. http://portalgeo.rio.rj.gov.br/. Accessed 2 December 2013.

[25] Forattini OP, Gomes A, Santos JLF, Kakitani I, Marrucci D. Culicidologia Médica. São Paulo. São Paulo: ‘EDUSP; 2002.

[26] Forattini OP, Gomes Ade C, Santos JL, Kakitani I, Marucci D (1990). Frequency in human environment and dispersion of Culicidae mosquitoes in an area adjacent to a primitive Atlantic plain forest. Rev Saude Publica.

[27] Honorio NA, Silva Wda C, Leite PJ, Goncalves JM, Lounibos LP, Lourenco-de-Oliveira R (2003). Dispersal of *Aedes aegypti* and *Aedes albopictus* (Diptera: Culicidae) in an urban endemic dengue area in the State of Rio de Janeiro, Brazil. Mem Inst Oswaldo Cruz.

[28] IBGE, Brazilian Institute of Geography and Statistics. Censo demográfico 2010: aglomerados subnormais: informações territoriais. http://bibliotecca.ibge.gov.br/visualizacao/periodicos/552/cd_2010_agsn_if.pdf. Accessed 21 January 2013.

[29] Center for Disease Control and Prevention. Epi Info, 2010. http://www.cdc.gov/epiinfo/downloads.htm. Accessed 21 January 2013.

[30] Grieve RB, Glickman LT, Bater AK, Mika-Grieve M, Thomas CB, Patronek GJ (1986). Canine *Dirofilaria immitis* infection in a hyperenzootic area: examination by parasitologic findings at necropsy and by two serodiagnostic methods. Am J Vet Res.

[31] Bowman DD (2012). Heartworms, macrocyclic lactones, and the specter of resistance to prevention in the United States. Parasit Vectors.

[32] Atkins CE, Murray MJ, Olavessen LJ, Burton KW, Marshall JW, Brooks CC (2014). Heartworm ‘lack of effectiveness’ claims in the Mississippi delta: computerized analysis of owner compliance--2004–2011. Vet Parasitol.

